# Breaching the Castle Walls: Hyaluronan Depletion as a Therapeutic Approach to Cancer Therapy

**DOI:** 10.3389/fonc.2015.00192

**Published:** 2015-08-28

**Authors:** H. Michael Shepard

**Affiliations:** ^1^Halozyme Therapeutics Inc., San Diego, CA, USA

**Keywords:** hyaluronan, extracellular matrix, tumor microenvironment, hyaluronidase, treatment resistance, interstitial fluid pressure, PEGPH20

## Abstract

Hyaluronan (HA) has many functions in the extracellular milieu of normal and diseased tissues. Disease-associated HA accumulation has been shown to predict a worsened prognosis in cancer patients, with tumors having a high-extracellular HA content (HA-high) being more aggressive than their HA-low counterparts. HA-high tumor aggressiveness is derived from the specialized biomechanical and molecular properties of the HA-based assembly of HA binding proteins and the growth-promoting factors that accumulate in it. Biophysical characteristics of an HA-high tumor microenvironment include high tumor interstitial pressure, compression of tumor vasculature, and resulting tumor hypoxia. Within the tumor cell membrane, HA receptors, primarily CD44 and RHAMM, anchor the HA-high extracellular network. HA–CD44 association on the tumor cell surface enhances receptor tyrosine kinase activity to drive tumor progression and treatment resistance. Together, malignant cells in this HA-high matrix may evolve dependency on it for growth. This yields the hypothesis that depleting HA in HA-high tumors may be associated with a therapeutic benefit. A pegylated form of recombinant human hyaluronidase PH20 (PEGPH20) has been deployed as a potential cancer therapeutic in HA-high tumors. PEGPH20 can collapse this matrix by degrading the HA-assembled tumor extracellular framework, leading to tumor growth inhibition, preferentially in HA-high tumors. Enzymatic depletion of HA by PEGPH20 results in re-expansion of the tumor vasculature, reduction in tumor hypoxia, and increased penetration of therapeutic molecules into the tumor. Finally, HA-depletion results in reduced signaling via CD44/RHAMM. Taken together, HA-depletion strategies accomplish their antitumor effects by multiple mechanisms that include targeting both biophysical and molecular signaling pathways. Ongoing clinical trials are examining the potential of PEGPH20 in combination with partner therapeutics in several cancers.

## Introduction

Hyaluronan (HA) is a large, unbranched, glycosaminoglycan that consists of repeating disaccharides of d-glucuronic acid and *N*-acetylglucosamine (1). HA is commonly referred to as a ubiquitous structural component of the extracellular, pericellular, and intracellular matrices, often with the function of lubricating surface-to-surface movement of joints or muscle (2). That characterization, although correct, does not convey the importance of the role that HA, together with its partner proteoglycans [the HA binding proteins (HABPs, or hyaladherins)], play in regulating cell behavior. HA in solution is polyanionic, conferring a largely negatively charged molecule in solution. Extensive hydration and the polyanionic nature of HA allow it to expand and occupy a large hydrodynamic volume (3, 4). High concentrations of HA, or HA combined with HABPs, creates a size-selective barrier in which small molecules can diffuse freely but larger molecules are partially or completely excluded (5). The biophysical properties of HA are exploited by tumor cells to create a sanctuary that is protective with respect to systemic therapies as well as host immune surveillance, as will be discussed below. Overall, the accumulation of HA in tumors can result in more aggressive malignancy (6–15). This review provides a brief overview of selected functions of HA and discusses the possible mechanisms through which accumulation of HA can make a tumor more aggressive and the mechanisms through which HA depletion can improve therapy of cancer.

## Hyaluronan: A Key Component of the Tumor Microenvironment and Promising Therapeutic Target

### Hyaluronan and its binding partners

**Figure 1:**
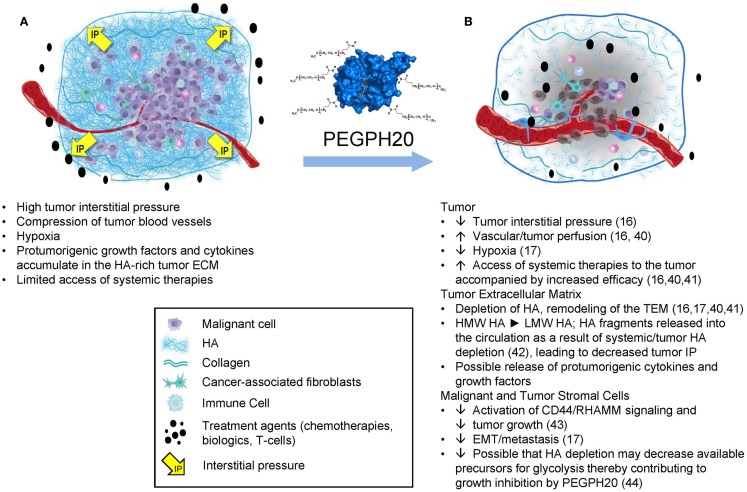
**The impact of HA depletion from a tumor with an HA-high phenotype**. **(A)** An HA-high tumor, encompassed by a fibrous capsule. As HA accumulates in the tumor it adsorbs water, resulting in expansion of the tumor stroma, which is limited by the fibrous capsule, resulting in increased tumor interstitial pressure, collapse of tumor-associated vasculature, and other sequelae as shown. **(B)** After treatment with PEGPH20, high-molecular weight HA is degraded to fragments, which diffuse into newly expanded vasculature, resulting in a dose-dependent normalization of tumor interstitial pressure and other changes, which result in tumor growth inhibition and increased access to systemic therapies. Abbreviations: ECM, extracellular matrix; HA, hyaluronan; PEGPH20, pegylated recombinant human hyaluronidase; pO2, partial pressure of oxygen; VEGF, vascular endothelial growth factor. Figure adapted from Ref. (45). Data from Ref. (16, 17, 40–44).

The properties of HA that clearly affect tumor progression include its biophysical properties, which lead to accumulation of large amounts of water into the tumor microenvironment (TME) as a result of its hygroscopic nature as well as its net negative charge, which induces the molecule to take on an expanded volume of hydration (4). HA molecules within the TME absorb a substantial number of water molecules (~15 per disaccharide) causing the extracellular matrix (ECM) to swell, resulting in high tumor interstitial pressure (tIP), collapse of the tumor vasculature, and tumor hypoxia (16, 17). In addition, as HA is extruded into the TME by tumor cells, it also coordinates the assembly of a complex ECM through its ability to bind an array of HABPs (6, 18). Evidence from *in vitro* studies suggests that high expression of HAS3 induces abundant cell surface microvilli, which vastly increases the surface area of the tumor cell. Tumor cells within a tumor with high-HA accumulation (HA-high) are networked into the ECM by the HA receptors on their surface. The best characterized interaction is between HA and its principal receptor, CD44. HA *in vitro* forms three-dimensional pericellular coats and can also form cables that may be involved in cell-to-cell communication (19). The HA-high matrix provides scaffolding for many HABPs (6, 18, 20). The matrix structural HABPs are primarily proteoglycans, and the most common side chains are composed of chondroitin sulfates (6, 18). Some HABPs [e.g., pentraxin-3, together with inter-alpha trypsin inhibitor and tumor necrosis factor-stimulated gene-6 (TSG-6)] serve to cross link the HA-high tumor ECM (21). Higher levels of pentraxin-3 expression are associated with a more aggressive disease in pancreatic cancer patients (22). The chondroitin sulfates add to the elevated tIP because they coordinate water molecules like HA, and their dense negative charge can bind and store growth factors, which creates a reservoir of tumor-promoting molecules within the TME (23). However, in our work, the antitumor response to polyethylene glycol-conjugated (pegylated) recombinant human hyaluronidase PH20 (PEGPH20) correlates very closely to the HA content of a tumor (24). The result of these interactions in an HA-high tumor is a multidimensional and gel-like structure comprising malignant cells, fibroblasts, and immune cells, all tied up together by HA and its binding partners in a setting of high tIP (Figure 1A). The high tIP characteristic of HA-high tumors may have other biomechanical signaling consequences promoting tumor progression (25, 26).

Aside from creating a unique TME structured around HA, cells that overproduce HA have additional properties. Recently, Tammi and colleagues described the formation of tumor cell membrane protrusions that accompany the overexpression of HAS3, one of three enzymes that synthesize and secrete HA (27). These microvilli, which can spontaneously break away from the tumor cells, retain the HAS3 enzyme and are coated in HA. Because they are coated with HA, they have the potential to bind/activate CD44/RHAMM on other cells, including stromal cells. Such membrane vesicles could be an important messaging system from a HA-high tumor that could affect the behavior of other cells, locally or systemically (28).

The HA-high tumor may have additional advantages. In particular, the HA pericellular coat can physically inhibit the ability of immune cells to form synapses and kill malignant cells *in vitro* (29). The extension of these *in vitro* findings to *in vivo* models has shown that in HA-high tumors, there is reduced access for either monoclonal antibody (mAb; trastuzumab) or natural killer (NK) cells (30). Depletion of HA from the tumor enables access and leads to improved efficacy, making HA a target for improving the efficacy of mAb or immune cell therapy of cancer (31).

Hyaluronan not only provides structural support to the tumor ECM but also interacts with cell surface receptors CD147/RHAMM and CD44. Both CD44 and RHAMM expression are tightly controlled by the wild-type p53 tumor suppressor gene (32, 33), provoking the hypothesis that their dysregulation contributes to tumor progression (33, 34). Activation, or amplified signaling, of multiple tyrosine kinases is mediated by HA binding to its receptors CD44 and RHAMM (33–36). Because elevated tyrosine kinase in tumor cells leads to resistance to macrophage killing, this could be an important pathway for HA/CD44 tumor cells to escape immune surveillance (37). In addition to the effects on tumor progression described above, further studies are underway to investigate the global reduction of growth factor signaling that is expected in tumor cells following depletion of HA (36, 38, 39).

### Rationale for targeting HA in cancer

Hyaluronan accumulates in the ECM of many solid tumors, and with a very high frequency (87%) in pancreatic ductal adenocarcinoma (PDA) (6, 13, 40). Several key factors provide a strong rationale for targeting HA in the TME (Figures 1A,B) (16, 17, 40–45). Tumors that accumulate a relatively high amount of HA (the HA-high phenotype) have been shown to be more aggressive in mouse models and among cancer patients (13, 15, 16, 24). High HA production is sufficient for induction of epithelial-to-mesenchymal transition (EMT) and acquisition of a highly malignant and migratory/invasive phenotype both in normal and transformed epithelial cells (46–48). In the genetically engineered *Kras^LSL-G12D/^*^+^*;Trp53^LSL-R172H/^*^+^*;Cre* (KPC) mouse model of PDA, HA deposition is seen early in the tumorigenesis process and persists during tumor progression and metastasis (41). Similar observations (conservation of the HA-high phenotype) have been reported in primary and secondary lesions from breast cancer patients (49).

### Breaching the HA barrier

Hyaluronidases function to degrade HA in a variety of tissue types and physiologic settings. The potential for HA to be an antitumor target was first pursued by Baumgartner and colleagues (50–52), who conducted several clinical studies with partially purified animal-derived PH20. The Baumgartner hypothesis was that HA in tumors impeded the tumor cell exposure to therapy. Although the investigators reported positive results from early trials, a randomized trial done in high-grade astrocytomas with combined chemotherapy and radiation therapy with and without hyaluronidase did not find an advantage in the PH20 group (51). Although additional clinical studies were not pursued at that time, our knowledge of the biology of HA has progressed significantly since these early investigations, and an image of how HA accumulation enhances tumor progression has emerged (previously discussed). In addition, the availability of purified recombinant human PH20 (rHuPH20) has enabled exploration of a means to use the human enzyme for chronic, systemic therapy (53). Both rHuPH20 and PEGPH20 were initially compared for clinical suitability through pharmacokinetic studies in mice, which showed that rHuPH20 had a very short half-life (<3 min), while PEGPH20 was shown to have an extended half-life (10.3 h) *in vivo* (16). The pegylated molecule has been taken forward in explorations of utility in cancer through both preclinical studies and clinical studies. Preclinical animal model studies using HA-high and HA-low tumors were performed, all with the conclusion that tumors with the HA-high phenotype are more sensitive to PEGPH20 (16, 17, 24).

**Figure 2:**
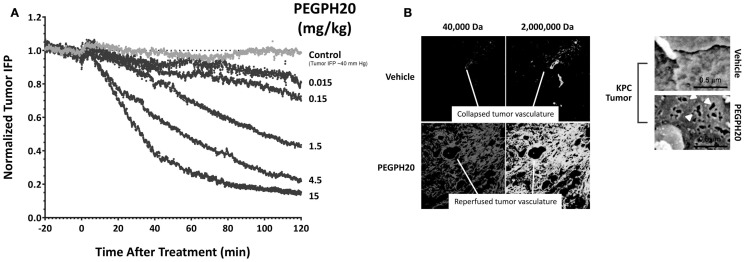
**Enzymatic HA depletion leads to normalization of tIP and normalization of tumor vasculature**. **(A)** PEGPH20 rapidly normalizes tIP. Dose-dependent effect of PEGPH20 [0 (control), 0.015, 0.15, 1.5, 4.5, 10, and 15 mg/kg] on tumor tIP in HA-high PC3 prostate tumors over a 2-h period following intravenous administration (starting tIP ~40 mm Hg). Abbreviations: HA, hyaluronan; IFP, interstitial fluid pressure; min, minutes; PEGPH20, polyethylene glycol-conjugated (pegylated) human hyaluronidase PH20; tIP, tumor interstitial pressure. Adapted with permission from Thompson et al. (16) **(B)** PEGPH20 treatment leads to vascular expansion and formation of endothelial fenestrae. Representative fluorescent images of KPC tumor from vehicle-treated (top left panels) and PEGPH20-treated (bottom left panels) mice (*n* = 4 mice for each cohort). Scanning electron microscopy images of pancreatic blood vessels in KPC (upper two right panels) mice following treatment with either vehicle or PEGPH20 (*n* = 4 mice for each cohort) reveal endothelial fenestrations (white arrowheads) only in the tumor microvasculature. PEGPH20-treated KPC mice. Endothelia in the healthy pancreata of control PC mice are comparable to the untreated KPC tumor. Abbreviations: HA, hyaluronan; KPC, *LSL-Kras^G12D/^*^+^*; LSLTrp53^R172H/^*^+^*; Pdx-1-Cre*; PC, *LSL-Trp53^R172H/^*^+^*; Pdx-1-Cre*; PEGPH20, polyethylene glycol-conjugated (pegylated) recombinant human hyaluronidase PH20. Adapted with permission from Jacobetz et al. (40).

The mechanism(s) through which HA-depletion results in tumor growth inhibition are still under investigation. Current data show that tumor perfusion by systemic therapies is increased following HA depletion by PEGPH20, resulting in a reversal of hypoxia and inducing other changes in the TME (16, 17). Accumulation of HA has been shown to be associated with loss of plasma membrane E-cadherin and β-catenin, suggesting disruption of adherens junctions, and increased potential for EMT, which is a predicate for the metastatic phenotype (54). PEGPH20 decreased the expression of hypoxia-related proteins and induced translocation of E-cadherin and β-catenin to the plasma membrane *in vivo* (17). Translocation of E-cadherin was also seen in tumors from a transgenic mouse model of pancreatic cancer and in a human non-small cell lung cancer sample from a patient treated with PEGPH20 (17). In conclusion, HA accumulation promotes tumorigenesis in multiple animal models and in many types of malignancies (15–17, 24, 27, 40, 41). HA depletion reverses these changes and suppresses tumor growth. We hypothesize that the antitumor effects of PEGPH20 are likely due to these major changes in the TME. However, a direct effect on tumor cells is possible as a result of the generation of low-molecular weight (LMW)-HA fragments that can compete with high-molecular weight (HMW)-HA for binding and activation of HABPs, including the HA receptors, CD44 and RHAMM (43). Literature describing the opposing roles of HMW-HA and LMW-HA is straightforward in cases in which there may be a high molar excess of LMW-HA, leading to antagonism of HMW-HA binding to hyaladherins and destabilization of the HA-mediated ECM. Other work suggests that accumulation of LMW-HA could lead to disruption of endothelial cell:cell interactions, and even to induction of inflammatory cytokines (43, 55). It is important to keep these proposed functions of LMW-HA in mind, as they could point to a toxicity resulting from systemic therapy with hyaluronidase. Studies in the KPC mouse model of PDA have shown that PEGPH20 treatment of tumor-bearing animals results in inhibition of tumor growth and a tumor-specific accumulation of chemotherapy (doxorubicin, gemcitabine), resulting in increased efficacy of these agents (40). Further studies have shown that increased tumor access to mAbs and immune cells can also occur following HA depletion. *In vitro* studies demonstrated that PEGPH20 treatment enhanced the access of trastuzumab and NK cells to HA-high tumors, and thereby increased both trastuzumab- and NK-cell-mediated tumor growth inhibitions (31). The mechanism leading to this tumor-selective increase in perfusion is the rapid reversal of high tIP (Figure 2A) (16), which results in not only vessel reperfusion but also the formation of fenestrae in the tumor vascular endothelium (Figure 2B) (40). A similar mechanism may explain the increased accumulation of *Salmonella* vector encoding an immune-stimulating cargo in KPC tumor-bearing mice (56).

The depletion of HA from the tumor ECM is likely to have a role in the antitumor effects of other coadministered potential therapeutics. For example, inhibitors of HA synthase (4-methylumbelliferone and methyl-beta-cyclodextrin) have been shown to have antitumor effects and can enhance the perfusion of chemotherapy into tumor vasculature (28, 57–59). Other agents that have been shown to affect tIP, but are not yet linked to HA loss, include the taxanes (60). Another recent report suggests that calcipotriol, a synthetic derivative of calcitriol (1,25-dihydroxyvitamin D3), can enhance the delivery of chemotherapy to pancreatic tumors through stromal remodeling (61). It is possible that the anti-inflammatory properties of calciferol are responsible for this result. Inflammatory signals are known to enhance HA synthesis, and result in HA accumulation and increased tIP (62). Suppression of inflammatory signals by calciferol is proposed to down-regulate the production of these mediators, which would then lead to decreased HA accumulation. Although there are agents other than PEGPH20 that reduce tIP and may also lead to decreased HA accumulation, none have been shown to induce the rapid reperfusion and formation of vascular fenestrae observed with PEGPH20.

The role of hyaluronidases in tumor biology has long been an area of investigation. The PH20 hyaluronidase is unique in that it is active with respect to degrading HA at both low pH and neutral pH. Other hyaluronidases (HYAL1, HYAL2, HYAL3, HYAL4) have been studied less extensively than PH20 (63, 64). From among these enzymes, HYAL1 has been proposed to promote tumorigenicity under certain conditions (65, 66). The mechanism(s) by which HYAL1 can promote tumorigenicity are still unclear. However, it is possible that the localized presence of HYAL1 in a tumor with the HA-high phenotype could be a mechanism to feed the increased dependence of tumor cells on glucose by facilitating the recycling of HA for cellular metabolism via the glycolysis pathway (67, 68), a process it may share with membrane associated HYAL2 (69). Therefore, long-term treatment with PEGPH20 might cause metabolic stress for tumor cells by depleting their supply of glucose precursors through removal of extracellular HA. In view of the reported tumor-promoting actions of Hyal1, it is also important to note that preclinical models have consistently shown a reduction in metastatic spread with PEGPH20 treatment (6, 41). These data serve to further highlight the differences between the various hyaluronidases and provide additional rationale for clinical development of PEGPH20. A summary of the proposed mechanisms of action for PEGPH20 enzyme therapy of cancer is shown in Figure 1B (16, 17, 40–45).

## Conclusion and Future Directions

The correlation between excess HA accumulation in the stroma of solid tumors (including breast, prostate, lung, and pancreatic cancers) and poor prognosis and short survival has been demonstrated. Numerous preclinical studies have described a role for HA in the growth and metastasis of solid tumors. Potential effects of HA accumulation include shielding cancer cells from immune cell attack and from antineoplastic therapies through a variety of mechanisms. Early phase clinical trials have demonstrated the benefits of adding PEGPH20 to chemotherapy for advanced pancreatic cancer. The results of a phase 1b clinical trial with PEGPH20 together with gemcitabine in pancreatic cancer patients has shown promising signs of efficacy (70). A companion diagnostic [derived from the tumor necrosis factor-stimulated gene-6 (TSG6) HABP] is being co-developed with PEGPH20 (71), which should help to identify patients most likely to benefit from PEGPH20 therapy by assessing pretreatment biopsies and monitoring the effects of treatment. Ongoing clinical trials of PEGPH20 will provide a further understanding of the clinical benefit of this agent as an adjunct to standard therapies in advanced cancer. Two clinical trials to evaluate PEGPH20 are currently enrolling patients: a randomized phase 1B/2 study of PEGPH20 plus modified FOLFIRINOX (leucovorin calcium, 5-fluorouracil, irinotecan hydrochloride, and oxaliplatin) versus modified FOLFIRINOX alone in patients with stage IV pancreatic cancer (72), and a randomized phase 2 study of PEGPH20 in combination with nab-paclitaxel and gemcitabine in patients with stage IV pancreatic cancer (73). These studies will further elucidate the benefits of PEGPH20 as an adjunct to anticancer therapies in patients with advanced pancreatic cancer, and studies are planned to investigate this strategy in other solid tumors. PEGPH20 is an important potential anticancer therapeutic because of its inherent antitumor activity, and its potential to be paired with other therapeutic modalities to achieve greater efficacy.

## Conflict of Interest Statement

Dr. H. Michael Shepard is an employee of Halozyme Therapeutics Inc.

## Appendix

### Key Concepts

#### Hyaluronan (HA)

Also known as hyaluronic acid, HA is a large, unbranched, glycosaminoglycan that consists of repeating disaccharides of d-glucuronic acid and *N*-acetylglucosamine, and is a key polysaccharide component of the ECM. Excess accumulation of HA in the tumor stroma is associated with poor prognosis and short survival.

#### The tumor cell sanctuary

Cancers with the HA-high phenotype also have high tIP, which leads to tumor vascular collapse, hypoxia, and inherent drug resistance. Elevated tIP is also associated with increased metastatic capability. The presence of an HA-coat around tumor cells leads to activation of the CD44 receptor, and likely RHAMM, which enhances tumor growth, drug resistance, and metastasis.

#### Depletion of HA has an effect on the tumorigenic properties of an HA-high tumor

Several agents decrease HA from the tumor ECM. 4-methylumbelliferone and methyl-beta-cyclodextrin inhibit HA synthesis, and PEGPH20 directly depletes HA. The fact that all three agents selectively inhibit tumor growth in HA-high tumors supports the validity of HA as a target in HA-high cancers.

#### HA depletion from a tumor with the HA-high phenotype inhibits tumor growth by several mechanisms

(A) Reversal of high tIP inhibits tumor cell EMT; (B) Reperfusion of the tumor by systemic chemotherapy, mAb therapy, and immune cell therapy; (C) Blocks CD44-associated signaling; (D) Reduced tyrosine kinase signaling increases sensitivity to immune surveillance and inhibits tumor growth and metastasis.
